# Multiple gene targeting siRNAs for down regulation of Immediate Early-2 (Ie2) and DNA polymerase genes mediated inhibition of novel rat Cytomegalovirus (strain All-03)

**DOI:** 10.1186/s12985-020-01436-5

**Published:** 2020-10-27

**Authors:** Krishnan Nair Balakrishnan, Ashwaq Ahmed Abdullah, Jamilu Abubakar Bala, Faez Firdaus Abdullah Jesse, Che Azurahanim Che Abdullah, Mustapha Mohamed Noordin, Mohd Lila Mohd-Azmi

**Affiliations:** 1 Department of Pathology and Microbiology, Faculty of Veterinary Medicine, University Putra Malaysia, Selangor, Malaysia; 2 Department of Microbiology, Faculty of Applied Science, Taiz University, Taiz, Yemen; 3 Department of Medical Laboratory Science, Faculty of Allied Health Sciences, Microbiology Unit, Bayero University, Kano, Nigeria; 4 Department of Veterinary Clinical Studies, Faculty of Veterinary Medicine, Universiti Putra Malaysia, Selangor, Malaysia; 5 Department of Physics, Faculty of Science, Universiti Putra Malaysia, Selangor, Malaysia

**Keywords:** Cytomegalovirus, Combinations, Small interfering RNA, Replication, Gene expression

## Abstract

**Background:**

Cytomegalovirus (CMV) is an opportunistic pathogen that causes severe complications in congenitally infected newborns and non-immunocompetent individuals. Developing an effective vaccine is a major public health priority and current drugs are fronting resistance and side effects on recipients. In the present study, with the aim of exploring new strategies to counteract CMV replication, several anti-CMV siRNAs targeting IE2 and DNA polymerase gene regions were characterized and used as in combinations for antiviral therapy.

**Methods:**

The rat embryo fibroblast (REF) cells were transfected with multi siRNA before infecting with CMV strain ALL-03. Viral growth inhibition was measured by tissue culture infectious dose (TCID50), cytopathic effect (CPE) and droplet digital PCR (ddPCR) while IE2 and DNA polymerase gene knockdown was determined by real-time PCR. Ganciclovir was deployed as a control to benchmark the efficacy of antiviral activities of respective individual siRNAs.

**Results:**

There was no significant cytotoxicity encountered for all the combinations of siRNAs on REF cells analyzed by MTT colorimetric assay (*P* > 0.05). Cytopathic effects (CPE) in cells infected by RCMV ALL-03 had developed significantly less and at much slower rate compared to control group. The expression of targeted genes was downregulated successfully resulted in significant reduction (*P* < 0.05) of viral mRNA and DNA copies (dpb + dpc: 79%, 68%; dpb + ie2b: 68%, 60%; dpb + dpc + ie2b: 48%, 42%). Flow cytometry analysis showed a greater percentage of viable and early apoptosis of combined siRNAs-treated cells compared to control group. Notably, the siRNAs targeting gene regions were sequenced and mutations were not encountered, thereby avoiding the formation of mutant with potential resistant viruses.

**Conclusions:**

In conclusion. The study demonstrated a tremendous promise of innovative approach with the deployment of combined siRNAs targeting at several genes simultaneously with the aim to control CMV replication in host cells.

## Introduction

CMV often considerate as silent public health burden usually does not produce any symptoms after the infection. The prime reason was due to the ability of the virus to enter asymptomatic infection, once the virus replication was controlled by the host immune system [1, 2]. However, CMV can cause life-threatening condition in immunocompromised patients whom undergo transplant procedures, individuals diagnosed with AIDS and injured with third degree burn and elderly people to some extent [3–5]. Sadly, immature immune system of unborn child is prone to CMV infection causing complications such as microcephaly, mental retardation and hearing loss [6, 7]. The term RNAi is generally understood as a mean of evolutionary conserved mechanism during post-transcriptional silencing triggered by double stranded RNAs, in wide range of eukaryotic organisms. This system was initially discovered in nematode *Caenorhabditis elegans* [8]. Slowly, the usage of RNAi turned to be a broader wave of advance which has been employed as a treatment method for many infectious diseases, cancerous and nerve dysfunction disorders [9–15]. Introducing synthetic 21–23 nucleotides of siRNA could specifically inhibit the cellular mRNA in order to control the gene expression [16]. Up to date, various studies have been reported that delivering siRNA, to inhibit the viral replication and gene expression of growing number of human infectious viruses were promising [12, 17]. It was suggested that a greater focus on siRNA could produce interesting findings that account more for the siRNA utilization as antiviral strategy for future therapeutics. Growing research work proves that RNAi can inhibit or suppress most of the viruses despite RNA or DNA and whether single or double stranded structure. Combination of RNAi targeting different conserved regions is expected to function efficiently in reducing the chances of viral escape. For instance, in other mono-therapies, sequence specific RNAi facing problem where the virus escapes due to the mutant in target region resulting in growth of viral mutant [18–20]. This statement validated by few studies such as polio, HIV and HCV where the viral activity of single siRNA reported to decrease in efficiency [19–21]. Viruses evolve to escape from host RNAi by encoding “suppressors of RNAi silencing” (SRS) which is another disadvantage of single use of siRNA. SiRNA cocktail targeting different region of viral genome lowers the chances of viral mutant formation and prevent viral escape [22–25]. Nevertheless, besides virus infections, siRNAs can be utilized to silence multiple genes at a time which could benefit in treating brain tumorigenesis involving multiple genetic alterations and also targeting a suitable apoptotic gene as a treatment [26, 27]. In addition, the availability to maintain the wide array of cells including islets of Langerhans could provide a strong foundation to use siRNAs for diabetic studies [28, 29]. Therefore, exploring combinations of siRNAs as a potential therapeutic approach for Malaysia CMV isolate has gear up for the treatment destination [30]. Interestingly, this local strain (RCMV ALL-03) has been identified crossing the placenta infecting the pups making it suitable for congenital infection on animal model [31]. The availability of the complete genome sequence of this virus add on more advantages for pathogenicity study and antiviral development [32, 33]. Therefore, this study was conducted to screen combinations of siRNA for their effectiveness on inhibiting RCMV ALL-03 virus replication and gene expression.

## Material and methods

### Selection of siRNA combinations

The siRNA combinations were selected based on the successive percentage of individual siRNA inhibiting RCMV ALL-03 replication and gene expression (data not shown). The tested siRNA combinations were dpb + dpc, dpb + ie2b, dpc + ie2b and dpb + dpc + ie2b where dpb and dpc siRNAs targeting DNA polymerase region while ie2b targeting Immediate early 2 region. The sequences of designed siRNAs were displayed in Table 1.

**Table 1:** Sequence of RCMV ALL-03 specific siRNAs and their location within the genome (Accession number KP967684.1)

siRNA	Sequence (5′ → 3′)		Position in genome
	Sense	Antisense	
ie2b	CCGAAGCACUGGACAAGUAtt	UACUUGUCCAGUGCUUCGGat	149,515
dpb	CUAUAAGGGUAGAAUAACAAtt	UGUUAUUCUACCCUUAUAGtg	65,041
dpc	GCCUGAUCGUACGUAUGAAtt	UUCAUACGUACGAUCAGGCtg	66,534
Negative control	UUCUCCGAACGUGUCACGUtt	ACGUGACACGUUCGGAGAAtt	NA

Sequence of the scrambled siRNA (negative control) used in this study was included below

*NA* not applicable

### Cell culture, transfection and virus infection

Rat embryo fibroblast cells were propagated in 10% fetal bovine serum supplemented with DMEM nutrient solution. The cells were maintained with at 5% CO_2_ and 37 °C in an incubator. Prior to siRNA transfection, the cells were seeded on 6 well plates to achieve 70–80% of confluency level. After 24 h, combination siRNAs were transfected together with lipofectamine 3000 transfection reagent (1.5 μl/well). In each well, siRNAs were mixed to produce a final concentration of 300 pmol/well. Appropriate controls for treatment and non-treatment were included as well followed by twenty-four hours of incubation. Meanwhile, during the incubation, DMEM media without antibiotic was replaced at 6 h post transfection to avoid any contamination. Next, cells were infected with RCMV ALL-03 isolated from uterus and placenta of rats [30] at MOI of 1. The plates were incubated for 1 h and then the cells were washed with cold PBS followed by addition of fresh DMEM medium supplemented with 10% serum. The cells were then monitored continuously for CPE occurrence and the cultured cells were collected for all the analysis at appropriate time points. Ganciclovir was used as positive control and scramble siRNA as negative control for treatment groups while untreated cells as positive control and uninfected cells as negative control for non-treatment groups. Each siRNA combination was duplicated and was performed twice independently.

### Cellular viability assay

Cells with optimized density, 1 × 10^5^ were seeded in 96 wells plate supplemented with DMEM media with 10% FBS. The cells were incubated at 37 °C for 24 h. Once the cells are established for the treatment, the media was discarded, and the confluent cells were washed with cold PBS. The combination siRNAs was transfected and incubated at 37 °C without the infection of the virus. The plates were removed from incubator at 24, 48 and 72 h of time interval. Then, MTT assay was carried out to identify the cell viability. All the cells were washed twice with PBS followed by addition of (5 mg/ml) MTT (tetrazolium dye, 3-[4,5-dimethylthiazol-2-yl]-2,5-diphenyltetrazolium bromide) reagent and incubated for 4 h at 37 ºC. The MTT addition procedure was taken place in dark area and the plate was covered with aluminium foil during the 4 h incubation due to the sensitivity of MTT towards light. Later on, the MTT reagent was discarded carefully, and 200 μl of dimethyl sulfoxide was added to each well. Then, the plate was covered again with aluminium foil and leave for 20 min at room temperature. This is to make sure the formazon dye crystals to dissolve completely into purple color. Lastly, the results were obtained from the measured absorbance at 570 nm using a spectrophotometer microplate reader. Each assay was performed in triplicates and the cytotoxicity was calculated from an average of 3 replicates. The final results are presented as mean ± standard deviation.

### Titration of effective combination siRNAs via TCID 50

TCID50 was conducted to investigate the effects of combination siRNAs on controlling RCMV ALL-03 titer at different time intervals of post infection (day 10 and 18). Following the transfection of combination siRNAs, the REF monolayers were infected with MOI 1 RCMV ALL-03. TCID50 of each combination siRNAs transfected samples were analyzed and compared with TCID50 of non-transfected virus control group as previously reported [34]. All treatments were performed in triplicate and the results were expressed as mean ± SE.

### Apoptosis analysis by flow cytometry

Flow cytometry technique was employed to evaluate the percentage of viable cells, early apoptosis cells; late apoptosis cells and necrotic cells of combinations siRNA treated and control groups. The samples were prepared by Annexin V-FITC Apoptosis Detection Kit (Nacalai Tesque, Japan). First, the cells were washed twice using cold PBS followed by trypsinization to detach the cells from the culture plates. Briefly, 1 × 10^6^ cells/ml were resuspended in Annexin V Binding Solution (1×). Then, the 100 µl of suspension cells were incubated with 5 µL of Annexin V-FITC Conjugate and 5 µl of Propidium Iodide (PI) at room temperature. After 15 min, 400 µl of Annexin V Binding Solution (1×) was added to the sample solution and were analyzed with a FACSCalibur flow cytometer (BD, Bioscience, USA).

### Droplet digital PCR

Droplet digital PCR is an emerging third generation of nucleic acid detection methodology gaining an interest for absolute quantification of any target sequence without the needs of standard curve. Before the commencement of ddPCR experiment, primers and probe were designed targeting DNA poly region and were synthesized by IDT technologies following standard procedures. Selected primer sequences were CCGAAGTACCAGATTCAA (forward primer), GACGAGAGGGAGTATATT (reverse primer) and FAM-CGGACGGTGAACTCGTTTTT-TAMRA (Taqman probe). The experiment began with the preparation of reaction mix at 11 µl per reaction. After that, 1.98 µl of each forward and reverse primers are added into the reaction mix followed by 0.55 µl of probe. The samples were labelled as shown in Table 2. Then, 5.49 µl of DNase-free water was added. All components were then mixed up properly by pipetting up and down. Equally aliquot which was 21 µl were dispensed into each reaction tube. Then, 1 µl of DNA sample are added accordingly into the PCR tubes. The reaction were again mixed properly and be allowed to be equilibrated at room temperature for about 3 min. Once the reaction mixtures were ready, 20 µl of the PCR reaction mixture were then loaded into the sample well of a DG8™ Cartridge as well as 70 µl of Droplet Generation Oil into the oil wells. The cartridge loaded with sample was placed inside the Q × 200 Droplet Generator to partition the sample hence generating the droplet for 2 min. Then, the obtained droplets were carefully transferred into a clean 96-well plate. The plate was then sealed with foil by using the P × 1 PCR Plate Sealer. PCR thermal cycling was then proceeded, and the sealed 96-well plate was placed in the Q × 200 Droplet Reader in order to detect the positive and negative droplets. QuantaSoft™ Software was used to analyze the obtained results.

**Table 2:** Samples used in ddPCR setup

Labelling of the sample	Type of siRNAs sample
Sample 1	dpb + dpc
Sample 2	dpb + ie2b
Sample 3	dpc + ie2b
Sample 4	dpb + dpc + ie2b
Sample 5	GCV
Sample 6	Negative control
Sample 7	Untreated
Sample 8	Uninfected

### Cytopathic effect rate of RCMV ALL-03

The replication of virus can be identified indirectly by physical appearance changes of normal cells. It is also known as cytopathic effect (CPE) monitoring. In order to determine the efficacy of combination siRNAs, the transfected wells were monitored for virus induced CPE out to 18 days post infection by a using Nikon Eclipse TS100 Inverted Microscope (Nikon Instruments, Inc., New York, USA). All the images of combination siRNAs transfected as well other control groups were compared and recorded.

### Real time RT-PCR for treated samples

Gene expression study of combination siRNAs treated samples were carried out using optimized custom designed Taqman probes via CFX96TM real time PCR detection system (Bio-Rad, USA). Since combination siRNAs having two different gene targets: IE2 and DNA poly, each combination siRNAs were analyzed for both genes’ expression. All the primers and probes have been optimized and appropriate standard curve have been generated (Additional file 1: Figure S1). RCMV ALL-03 mRNA was extracted from the control and siRNA treated samples following manufacturer instruction by using a GENEzol™ TriRNA Pure kit (Geneaid, UK) with slight modifications. The RNA was finally eluted in 30 µl of RNase-free water and was maintained on − 70 °C for further analysis. The quantity and quality of extracted RNA were assessed by spectrophotometry using the Nanodrop 1000 spectrophotometer (Thermo Scientific™). Next, complementary DNA was synthesized using Tetro cDNA synthesis kit (Bioline, UK) on thermal cycler (BioRad, USA) following manufacturer’s instructions. The prepared cDNA was stored at − 20 °C for subsequent analysis. Finally, gene expression study of combinations siRNAs treated samples were carried out using designed Taqman probes via CFX96™ real time PCR detection system (Bio-Rad, USA). Specific primers and probe were used for each siRNA region as depicted in Table 3. To normalize the expression analysis, Glyceraldehyde 3-phosphate dehydrogenase (GAPDH) was used as housekeeping gene which served as internal control to quantify the mRNA expression in all the combination siRNAs treated and non-treated samples. The reaction was set up with cDNA as template with 400 nM concentration of each specific forward and reverse primers, 100 nM of probe, 1 × SensiFast probe Hi-Rox mix and appropriate nuclease free water. The cycling conditions was followed as for standard curve generation (data not shown) Mean quantitative cycle values (cq) were obtained from the triplicates and recorded using Bio-Rad, CFX manager software version 3.0 (Bio-Rad, USA).

**Table 3:** Primers and probe sequences used for real-time PCR assay

Target	Primer sequence	Product size (bp)
*GAPDH*		
Forward	tctccaccactatcgcagaa	100
Reverse	ttggcagcttggactatgct	
Probe	FAM-tccgttttggcagagaagatgcaa-TAMRA	
*ie2b*		
Forward	gcgattttgatctacgtg	256
Reverse	acagcgaacctatagaca	
Probe	FAM-caacggggggaggaaacaga-TAMRA	
*dpb*		
Forward	aggacatcatcacgagaa	100
Reverse	tcaaacaaagatagcggg	
Probe	FAM-agatcggtggggtatcagg-TAMRA	
*dpc*		
Forward	ccgaagtaccagattcaa	102
Reverse	gacgagagggagtatatt	
Probe	FAM-cggacggtgaactcgttttt-TAMRA	

### Data analysis for quantification of gene expression

Four combination siRNAs: dpb + dpc, dpb + ie2b, dpc + ie2b and dpb + dpc + ie2b were assessed for their effectiveness on inhibiting the IE2 and DNA poly gene expression of RCMV ALL-03. The relative fold change of the genes treated with siRNA were compared with untreated control group and the relative fold changes were calculated using − ΔΔCT method. Bio-Rad, CFX manager software version 3.0 (Bio-Rad, USA) was used for the normalization of target region with house-keeping gene GAPDH and quantification of fold changes were calculated as described previously [35].

### Calculating knockdown efficiencies

The percentage of knockdown efficiency was calculated following − ΔΔCT method with slight modification [36]. The levels of siRNA treated target expression was normalized to non-target GAPDH as reference gene (REF) within the same sample as equation below:

$$\Delta {\text{Ct}} = {\text{Ct}}_{{({\text{Target}})}} - {\text{ Ct}}_{{({\text{REF}})}}$$

Next, the ΔCt for each biologically replicate was transformed exponentially to the ΔCt expression followed by determining the standard deviation. The equation as follows:

$$\Delta {\text{Ct expression}} = {2}^{{ - \Delta {\text{Ct}}}}$$

Then, the mean was normalized to the expression of siRNA treated sample with non-targeting siRNA sample to find ΔCt expression level. Percentage of knockdown was calculated as equation below:

$$\% {\text{ Knock down}} = \left( {{1} - \Delta \Delta {\text{Ct}}_{{({\text{normalized}}\;{\text{expression}})}} } \right) \times {1}00$$

### Resistance of RCMV ALL-03 to siRNA combination treatment

Drug resistances are important issue to be addressed where virus has the capability of developing it. In this combination treatment, the ability of RCMV ALL-03 to develop drug resistance via mutation occurrence in target regions was investigated. After the siRNA combination transfection, RCMV ALL-03 was infected and the extracellular progeny virus was harvested after 14 days pi. The harvested virus was used to re-infect the REF cells after being transfected again with siRNA combinations. After 14 days pi, the virus was harvested, and this method was repeated up to 5 infections. The final harvested virus was subjected for nucleic acid extraction together with stock of virus. Upon confirmation, the PCR products were sent for sequencing and the obtained results were compared for sequence identity using Clustal Omega online software.

### Statistical analysis

All statistical analyses were performed using GraphPad Prism version 6 (GraphPad Software, USA). *P* values < 0.05 were considered significant. Results are expressed as mean ± SD from a representative experiment performed in triplicate.

## Results

### Effects of combination siRNAs on cell viability

Combinations of siRNAs were subjected for cell viability assay using MTT cell proliferation method. Cytotoxicity analyses of tested siRNA combinations demonstrated that cell viability was almost unaffected at an optimized concentration of 300 pmol at different time points. However, the triple combinations dpb + dpc + ie2b exhibit 50% toxicity level at 48 and 72 h for 300 pmol. On the other hand, all the double siRNA combinations such as dpb + dpc, dpb + ie2b and dpc + ie2b showed more than 70% of cell viability at highest concentration 300 pmol (Additional file 1: Figure S2).

### Small interfering RNAs inhibit virus growth

**Fig. 1:**
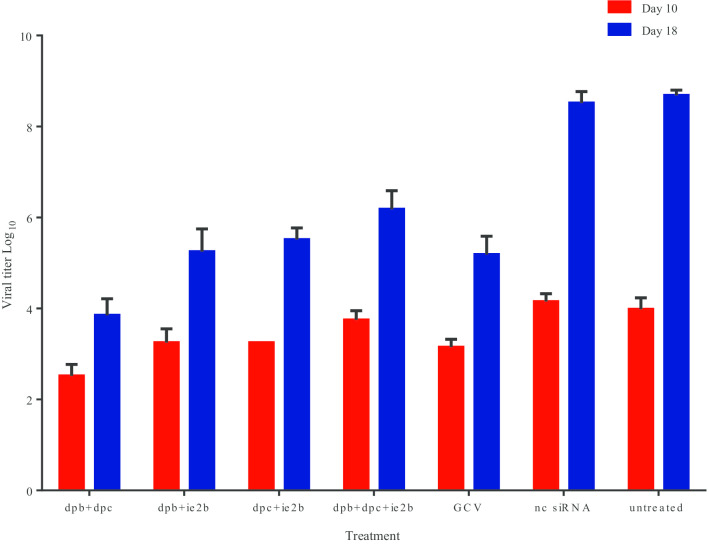
Results of a TCID50 assay to illustrate the effect of combinations siRNA on the yield of RCMV ALL-03 progeny virus in REF cells. The REF cells were transfected with combinations siRNAs at 300 pmol concentration, negative control siRNA and positive control GCV. At day 10 and 18 p.i, the plates were removed from incubator to calculate the TCID50 and the data represent the mean ± SD of 2 independent experiments

RCMV ALL-03 was silenced with double or triple combinations and the virus titers were quantified by TCID50. The reading of viral titer was recorded at two different days: 10 and 18 (Fig. 1). This is because no significant viral load was detected at day 10 and maximum growth of virus could be achieved at day 18 (Fig. 1). Overall, different combinations siRNA showed different level of virus inhibiting capacity which siRNAs targeting DNA polymerase showed better RCMV ALL-03 inhibition rate. Administration of the siRNAs before RCMV ALL-03 prevented the progression of the infection, since virus titers did not increase exponentially from day 10 pi to day 18 pi. By contrast, virus titers increased significantly from day 10 pi to day 18 pi in negative control siRNA and untreated groups. For instance, dpb + dpc (2.5 log_10_TCID50/ml, 3.8 log_10_TCID50/ml) showed better viral inhibition rate at day 10 and 18, respectively followed by dpb + ie2b (3.2 log_10_TCID50/ml, 5 log_10_TCID50/ml) and dpc + ie2b (3.2 log_10_TCID50/ml, 5.1 log_10_TCID50/ml). All these combinations had similar viral titer compared to positive control ganciclovir 3.2 log_10_TCID50/ml at day 10 p.i and 4.9 log_10_TCID50/ml at day 18 p.i. Therefore, the differences among the siRNA treated and ganciclovir are insignificant (*P* > 0.05). However, triple combinations dpb + dpc + ie2b (3.5 log_10_TCID50/ml, 5.9 log_10_TCID50/ml) displayed a lowest rate of viral inhibition compared to other combinations and GCV at both day 10 and 18. On the other hand, negative control siRNA and untreated groups showed higher viral titer than siRNA treated groups: 4 log_10_TCID50/ml at day 10 p.i and 8 log_10_TCID50/ml at day 18 p.i.

### Annexin V apoptosis study by flow cytometry

**Fig. 2:**
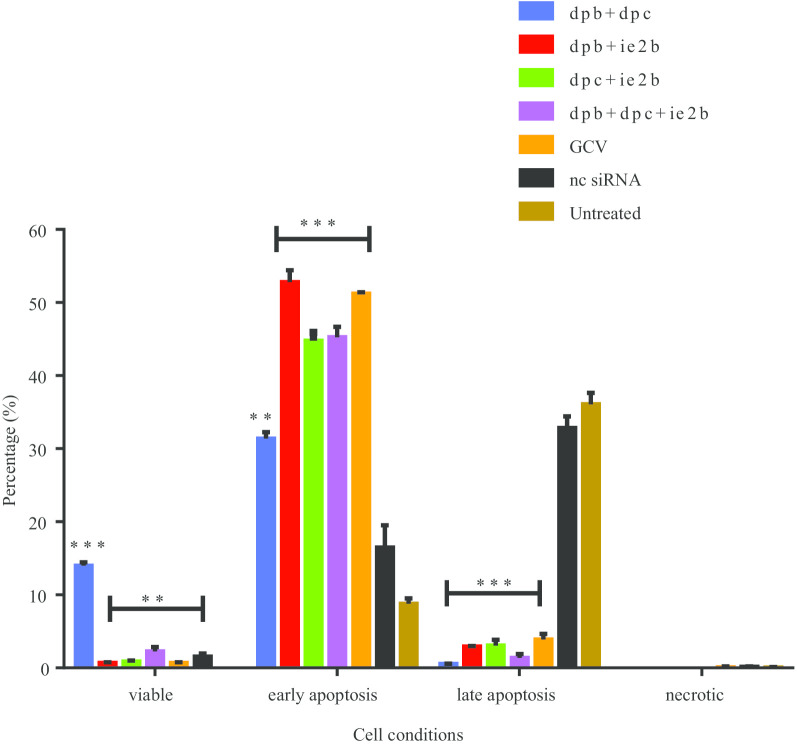
Percentage of various distribution of REF cell conditions. The percentage of viable, early apoptotic, late apoptotic and necrotic cells were analysed for all the siRNA treated group. Each data point represents the mean of three independent experiments ± SD. *Significantly different from the control (*P* < 0.05)

Analysis of RCMV ALL-03 infected cells undergoing apoptosis/necrosis upon combination siRNAs treatment were studied using flow cytometry. Dot plot view illustration was used to interpret the results of REF cells (Additional file 1: Figure S3). As revealed in Fig. 2, the percentages of viable cells are lesser compared to early apoptotic cells for all the siRNAs and GCV treated groups. Compared to untreated control group, dpb + dpc has significant number of viable cells and early apoptotic cells 13.6% ± 0.23 and 31.2% ± 0.17 respectively. The significant number of cells recorded in early apoptotic were all the combination siRNAs and GCV treated group where dpb + ie2b having the highest early apoptotic cells (53.4% ± 3.52). In addition, all the siRNAs and GCV treated groups showed least number of late apoptotic cells compared negative control siRNA and untreated groups. Meanwhile, lesser cells (0.1–2%) were observed for necrotic phase for all the treated groups including the control groups.

## Viral DNA particles quantification by DDPCR

### Quantification of DNA copies/µl

**Fig. 3:**
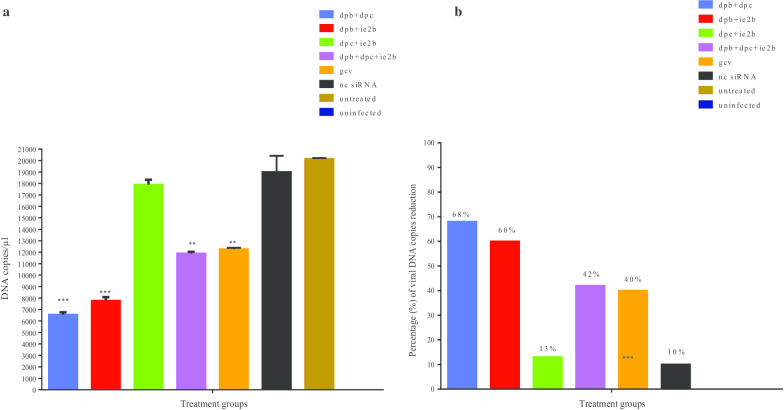
Results of RCMV ALL-03 absolute viral load quantification by droplet digital PCR. **a** The RCMV ALL-03 viral load (DNA copies/µl) in all the treatment groups (*P* < 0.005). **b** The percentage of RCMV ALL-03 DNA copies reduction compared to untreated control group

The quantification of RCMV ALL-03 DNA copies were presented per µl in the 100-fold diluted sample. Therefore, the total viral DNA copies/µl from stock template was calculated and presented in Table 4. Visualization of ddPCR analysis plot having positive and negative droplets were screened prior to viral DNA quantification (Additional file 1: Figure S4). The final results have revealed that out of four combination siRNAs, dpb + dpc has tremendously reduced the viral titre with 6,400 DNA copies/µl with 68% of reduction compared to untreated control group having 20,200 DNA copies/µl. As expected, both of these siRNAs targeting DNA polymerase gene region showed better viral inhibition rate compared to combination siRNAs targeting IE2 gene region. Next, dpb + ie2b followed by dpb + dpc + ie2b limits the virus particles with 60% and 42% rate effectiveness respectively (Fig. 3). However, dpc + ie2b showed to have the least effectivity rate 13% with 17,600 DNA copies/µl. In comparison with other siRNA treated groups, commercial drug GCV treatment was found to be median effective rate in reducing the viral DNA copies (12,200 copies/µl) with 40% of efficiency. Only 2% of difference in DNA copies reduction percentage was encountered between dpb + dpc + ie2b and GCV treatment groups. Overall, the percentage of viral reduction was apparently moderate for double and triple combination siRNAs and interestingly, triple siRNAs combination displayed lesser inhibition rate than double combination (dpb + dpc and dpb + ie2b). On the other hand, untreated control group is having high number of viral particles (20,200 copies/µl) due to the absence of any form of treatment.

**Table 4:** Total amount of DNA copies in stock template

Sample	Viral DNA copies in ddPCR mixture	Viral DNA copies per µl (100 fold diluted)	Total viral DNA copies/ µl from stock template	Percentage of viral DNA copies reduction (%)
dpb + dpc	3.2	64	6400	68
dpb + ie2b	4	80	8000	60
dpc + ie2b	8.8	176	17,600	13
dpb + dpc + ie2b	5.9	118	11,800	42
gcv	6.1	122	12,200	40
nc siRNA	9	180	18,000	10
Untreated	10.1	202	20,200	0
Uninfected	0	0	0	0

### Antiviral screening using CPE rate development

**Fig. 4:**
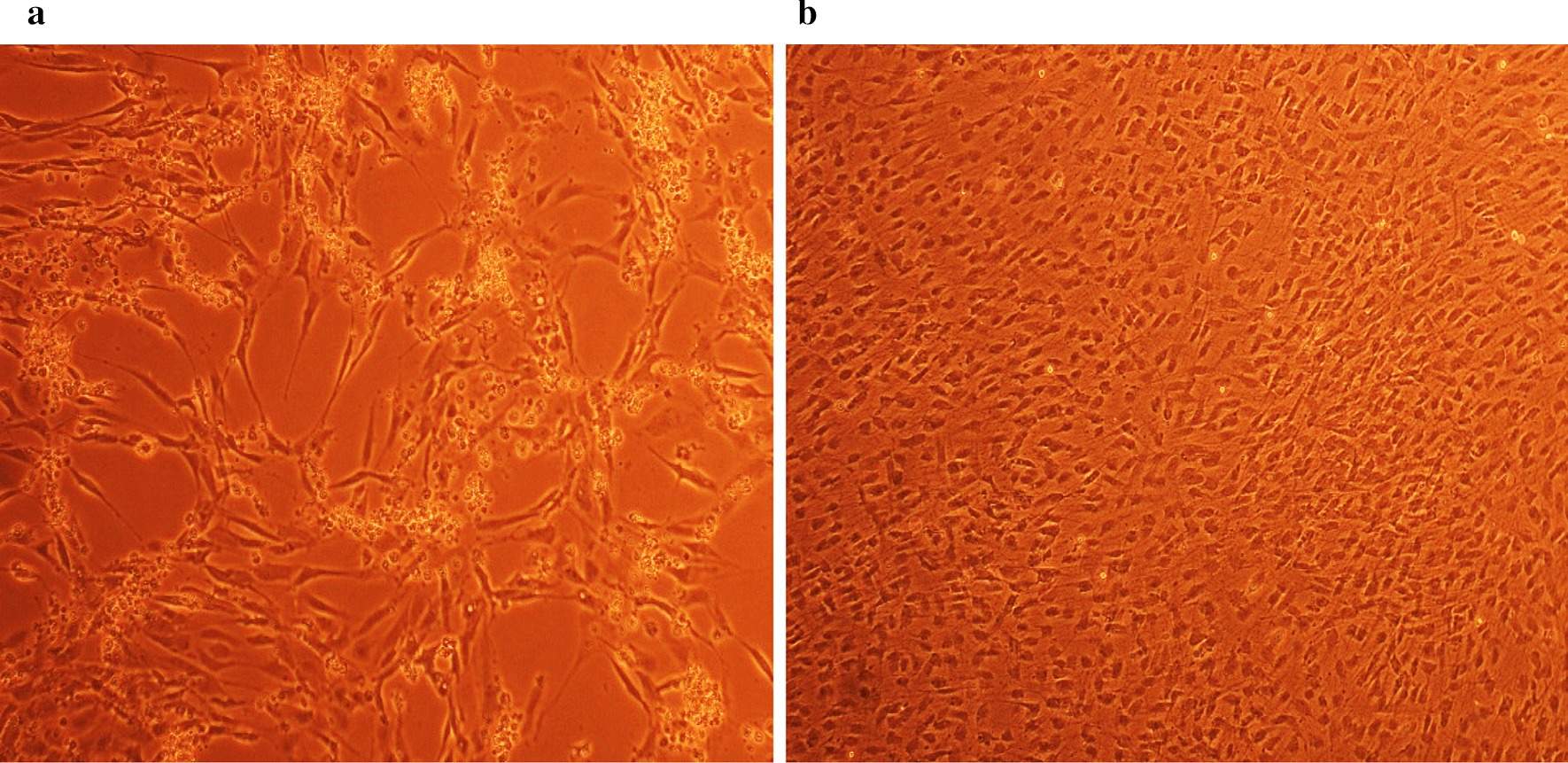
REF cells were transfected with dpb + dpc (**a**) and dpb + ie2b (**b**) combination siRNAs followed by RCMV ALL-03 infection. The virus induces specific CPE characterized by rounding of the cells, shrinkage, ballooning and destruction and detachment of cell layers. The cells were visualized at day 14 using an inverted microscope at 10 × magnification

**Fig. 5:**
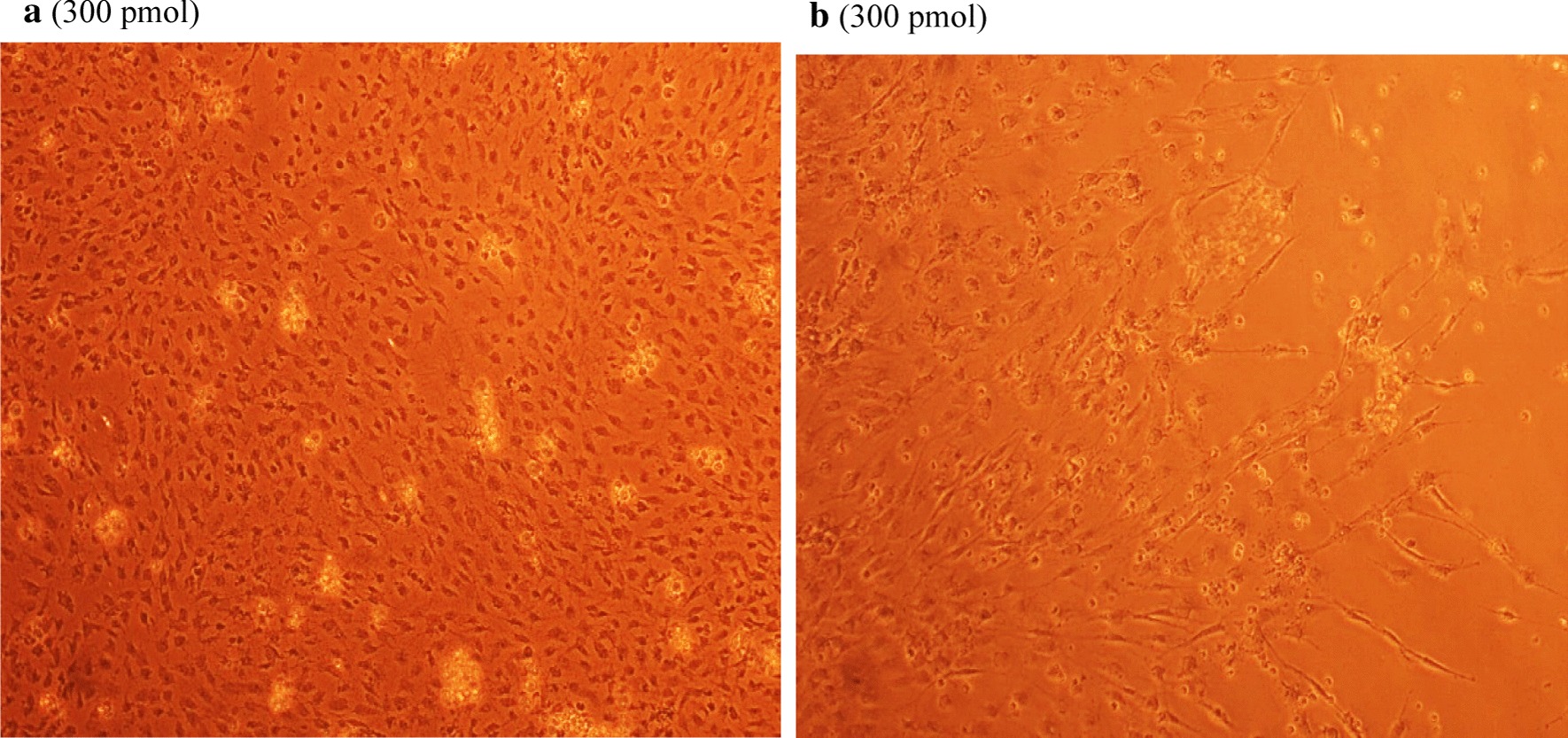
REF cells were transfected with dpc + ie2b (**a**) and dpb + dpc + ie2b (**b**) combination siRNAs followed by RCMV ALL-03 infection. The virus induces specific CPE characterized by rounding of the cells, shrinkage, ballooning and destruction and detachment of cell layers. The cells were visualized at day 14 using an inverted microscope at 10 × magnification

**Fig. 6:**
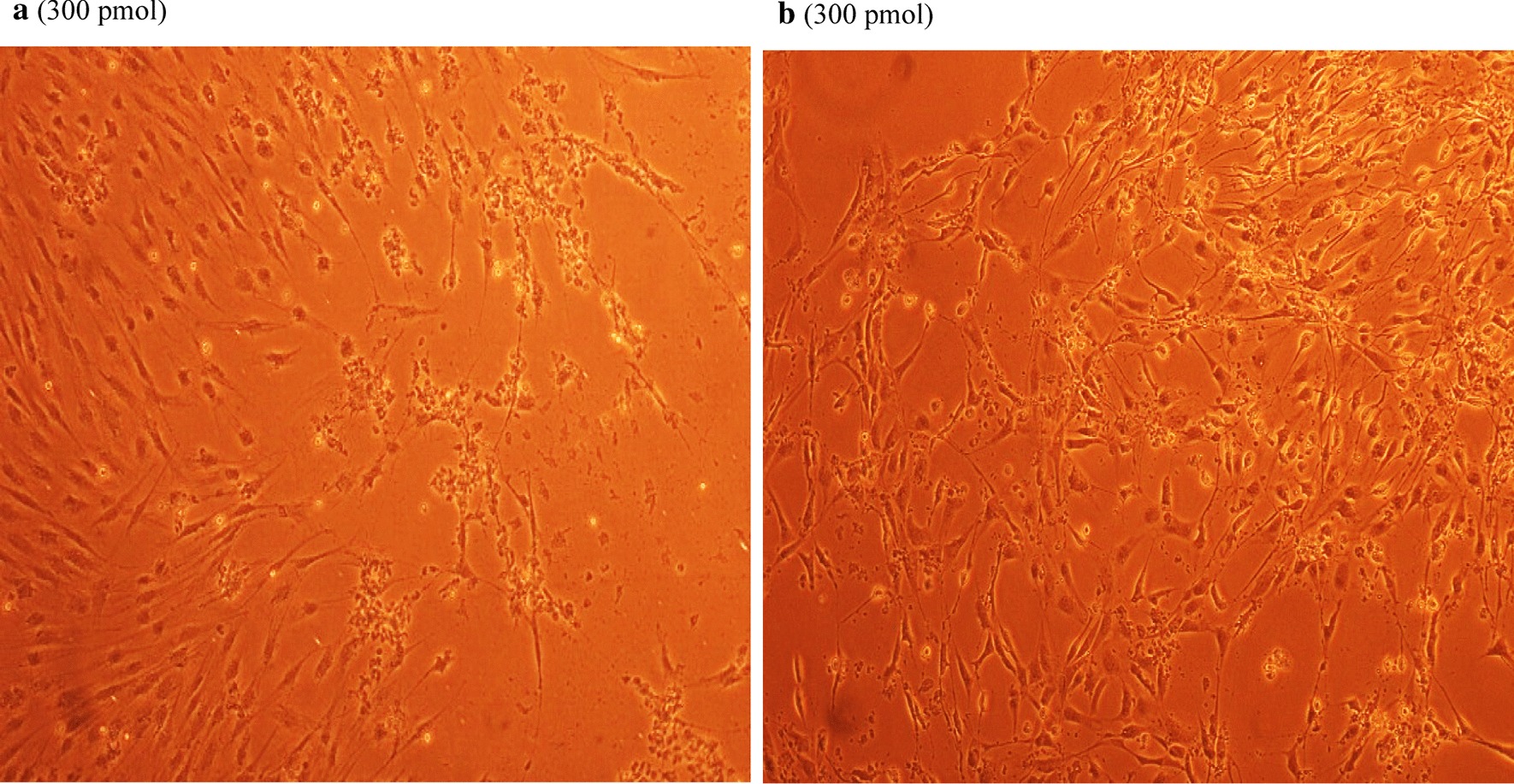
REF cells were transfected with GCV (**a**) and negative control siRNA (**b**) treated followed by RCMV ALL-03 infection. The virus induces specific CPE characterized by rounding of the cells, shrinkage, ballooning and destruction and detachment of cell layers. The cells were visualized at day 14 using an inverted microscope at 10 × magnification

**Fig. 7:**
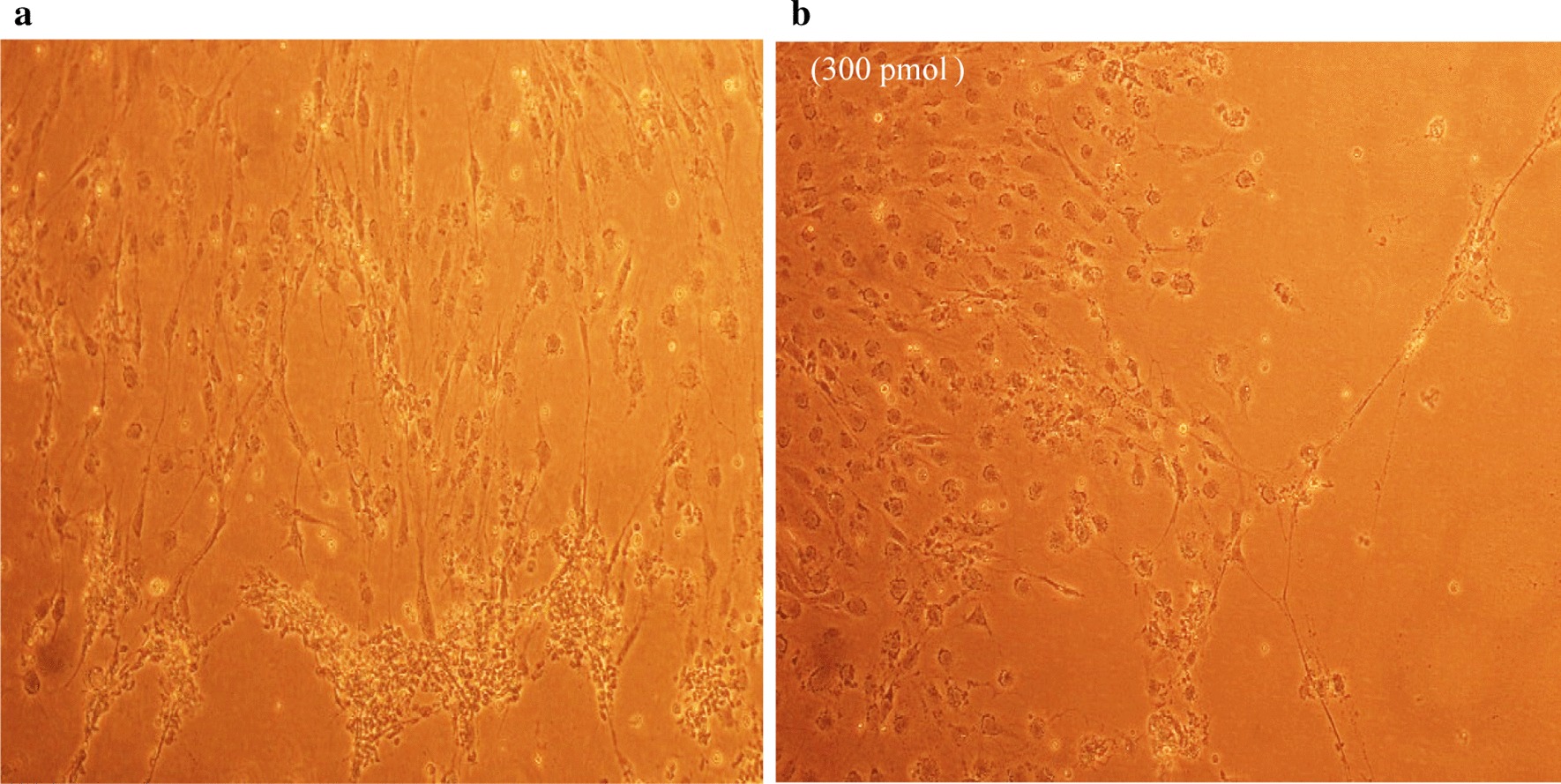
REF cells were transfected with untreated (**a**) and uninfected (**b**) followed by RCMV ALL-03 infection. The virus induces specific CPE characterized by rounding of the cells, shrinkage, ballooning and destruction and detachment of cell layers. The cells were visualized at day 14 using an inverted microscope at 10 × magnification

To study the CPE rate of RCMV ALL-03 in combination siRNAs treated and non-treated groups, REF cells were transfected with 300 pmol combination siRNAs followed by viral infection, subsequently the cells were monitored for morphological changes at day 14. Cytopathic effect is a reliable method to monitor the structural changes of host cells resulted from CMV invasion and propagation. There are distinct differences between CMV infected and non-infected groups. Normal cells retain their fibroblast shape, no plaque formation and tightly stuck to the plate up to day 14 (Fig. 4b). In contrast, the cells infected with RCMV ALL-03 exhibited evident morphological changes, starting from cell ballooning, cytoplasmic extension, shape changed to more elongated and finally forming plaque by detaching from flask. The continuation of plaque expands and achieves advanced CPE where most of the cells died and detach from the flask as shown in Fig. 4a. In order to assess the effectivity of combination siRNAs, all the treated cells were examined at day 14 and the rate of CPE development was recorded. As expected, all the combination siRNAs showed to protecting cells from RCMV ALL-03, however, the protection degree was differing from one and another. Out of four combinations, dpb + dpc shown to be most potent combination siRNAs where least rate of CPE was recorded (Fig. 5a). At day 14, 300 pmol of dpb + dpc was found to starting CPE with cell ballooning indication as showed in same figure. In comparison, negative control siRNA and virus control cells started to exhibit significant advanced CPE formation at day 14. Followed by dpb + dpc, dpb + ie2b and dpb + dpc + ie2b showed a good inhibition of CPE at day 14 (Figs. 5 and 6). Contrary to expectations, the results did not show a constant finding in all the siRNAs where dpc + ie2b siRNAs are less effective and displayed similar rate of CPE development with negative control siRNA and virus control cells proving their incapability in providing protection for host cells against RCMV ALL-03. These findings showed that triple combination siRNAs are not effective as double combination to inhibit the CPE rate. Thus, this proves the misconception theory of conveying more siRNAs combination providing better viral inhibition rate. The difference in results may be explained by the fact that siRNAs targeting different region of genome having different rate of CPE inhibition effectiveness. Taking into consideration as positive treatment control group, commercial drug GCV exhibited better rate of CPE inhibition (Fig. 7) compared to other combination siRNAs but lesser efficient than dpc + ie2b siRNAs.

### Real time gene expression analysis

**Fig. 8:**
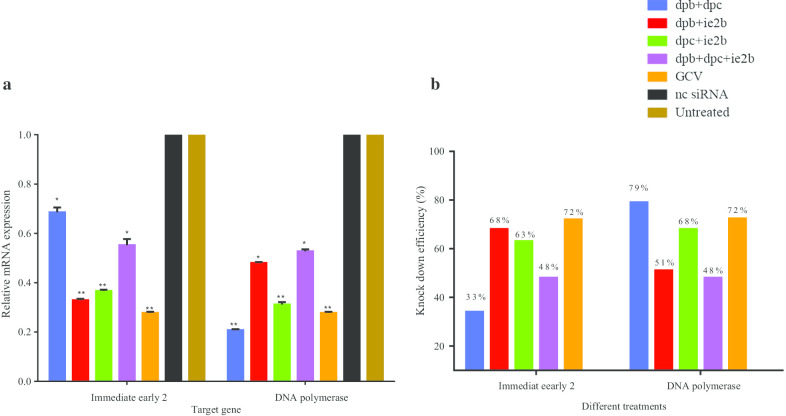
**a** Quantification of mRNA expression levels of different treatment groups. **b** The percentage of knockdown efficiency among siRNA treated and non-treated groups. The data shown are representative of three independent experiments at *P* < 0.05

In order to understand more on the role of each combination siRNAs during RCMV ALL-03 infection, all the four combination siRNAs targeting different regions were analyzed individually with specific primers. Overall, the results revealed that each combination siRNAs showed different degree of mRNA reduction with dpb + dpc, dpb + ie2b and dpc + ie2b showed a greater reduction compared to virus control group (Fig. 8a). SiRNA dpb + dpc found to be very effective by reducing the mRNA expression of DNA polymerase up to 79% (*P* < 0.05), however 39% for IE2 gene region. This is mainly due to the siRNAs itself where both siRNAs targeting only DNA polymerase and not IE2. Obvious differences were identified for the expressions of both IE2 and DNA polymerase for all the combination siRNAs. Fascinatingly, between the two genes, siRNAs targeting DNA polymerase found to have relatively lower mRNA expression compared to IE2. However, if compared to commercial drug GCV, the combination siRNAs were found to be less potent on inhibiting the IE2 and DNA polymerase expressions as shown in Fig. 8a. In comparison, triple siRNAs combination showed less mRNA inhibition ability on reducing the gene expressions compared to double siRNAs combinations.

### Knocking down efficiency

The silencing effects of siRNAs combination were further transformed to determine the percentage of knockdown efficiency as shown in Table 5 and Fig. 8b. Since the combinations are made up from different siRNAs targeting different regions, each combination were assessed for both IE2 and DNA polymerase genes knock down separately. For IE2 gene region, dpb + ie2b and dpc + ie2b combinations showed better knocking down efficiencies 63–68% compared to dpb + dpc and triple combination dpb + dpc + ie2b (33–48%) respectively. On the other hand, for DNA poly gene region, all the double siRNAs combination showed better knocking down percentage compared to IE2 region where the range percentage range were between 51 and 79%. The dpb + dpc combination targeting DNA polymerase alone showed best level of knocking down among siRNAs followed by dpc + ie2b and dpb + ie2b. This clearly shows that the designed siRNAs were found to working best on inhibiting DNA polymerase mRNA expression compared to IE2. Noticeably, triple combinations having 2 DNA poly siRNAs and one IE2 siRNA (dpb + dpc + ie2b) possess lesser potent on reducing the mRNA expression thus having a moderate knocking down efficiency compared to double siRNAs combination. Similar findings were also observed for viral quantification as well CPE rate observation. Therefore, these findings have proven again that more siRNAs in a combination not necessarily possess better knocking down efficiencies of a certain gene. Besides that, GCV still considered as better option in knocking down the both DNA polymerase and IE2 gene regions compared to rest of the siRNAs combination (Table 5). Another important finding was the knockdown efficiency of negative control siRNA. It was found to be zero percent of knocking down efficiency which are similar with virus control group, thus proving its nature as non-target control, which do not carry any impact on virus and host cells. In conclusion, each combinations siRNA hold different degree of gene knock down efficiency with dpb + dpc found to be high potent to knock down DNA polymerase gene while dpb + ie2b for IE2 gene.

**Table 5:** Percentage knockdown of IE2 and DNA polymerase gene expression

Treatment	ΔCt expression	ΔΔCt expression	% knock down
*A*			
dpb + dpc	0.094 ± 0.014	0.660 ± 0.104	33.90
dpb + ie2b	0.045 ± 0.001	0.318 ± 0.008	68.12
dpc + ie2b	0.051 ± 0.006	0.363 ± 0.046	63.67
dpb + dpc + ie2b	0.073 ± 0.008	0.518 ± 0.058	48.16
GCV	0.038 ± 0.001	0.270 ± 0.006	72.95
nc sirna	0.107 ± 0.014	1 ± 0.100	0
Virus control	0.142 ± 0.074	1 ± 0.518	0
*B*			
dpb + dpc	0.029 ± 0.005	0.208 ± 0.041	79.11
dpb + ie2b	0.069 ± 0.013	0.485 ± 0.097	51.46
dpc + ie2b	0.044 ± 0.011	0.315 ± 0.078	68.41
dpb + dpc + ie2b	0.073 ± 0.014	0.518 ± 0.103	48.14
GCV	0.038 ± 0.001	0.270 ± 0.006	72.95
nc sirna	0.107 ± 0.014	1 ± 0.100	0
Virus control	0.142 ± 0.074	1 ± 0.518	0

All the experiments were conducted twice independently in triplicates were presented as results ± SEM. The results were calculated using delta delta Ct method and normalized to housekeeping gene, GAPDH mRNA. (A) IE2 and (B) DNA polymerase

### Sequence identity for resistance of RCMV ALL-03 to siRNA combination treatment

**Fig. 9:**
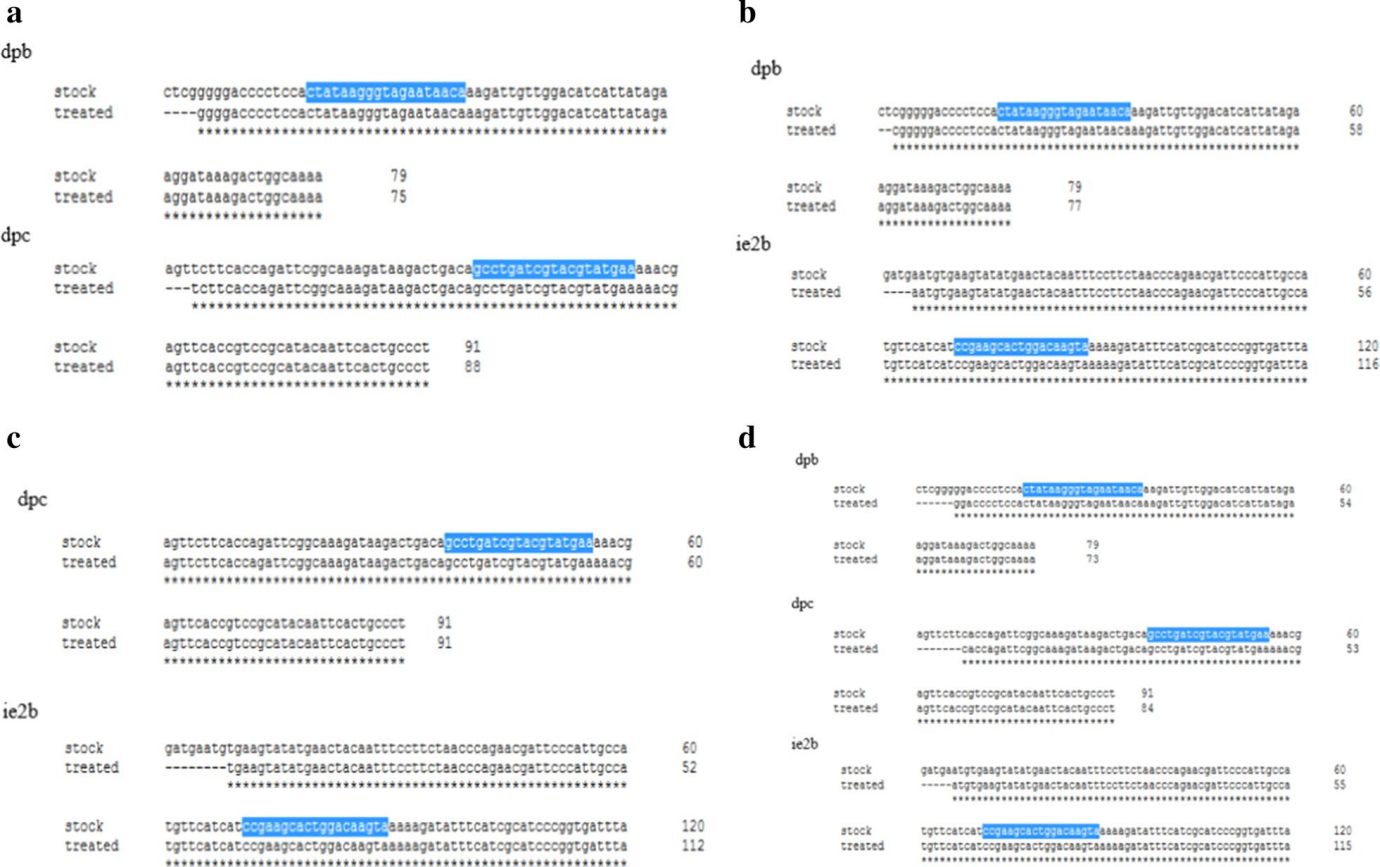
Alignment of DNA sequences from combiantion siRNAs treated group with stock virus for mutation identification. The siRNAs treated RCMV ALL-03 DNA was extracted and subjected for Sanger sequencing. The obtained results were aligned using online clustal omega software. The synthesized siRNA sequences was highlighted in blue. **a** dpb + dpc, **b** dpb + ie2b, **c** dpc + ie2b, **d** dpb + dpc + ie2b

It is common for viruses to develop resistance against treatment that subjected continuously over a certain period of time. To investigate the tendency to develop resistance via mutation by combination siRNAs treated RCMV ALL-0 were harvested. Then, the extracellular progeny virus was infected repeatedly up to 5 times on combination siRNAs treated REF cells. The final progeny virus was harvested and subjected for DNA extraction. The entire extracted DNA were sequenced by Sanger sequencer platform to identify any possible mutation occurrence and compared with stock RCMV ALL-03 solution using Clustal Omega online software system. For every combination siRNAs treatment, every region involved by the particular individual siRNA was sequenced to obtain all the possible occurrence of mutation. Remarkably, no any mutation was observed for all the siRNAs treated regions thus, proving the ability of designed siRNAs to not developing or delayed the development of siRNAs resistance by RCMV ALL-03. Currently, 5 passages of siRNA treated RCMV ALL-03 were sequenced and more passages needed to be sequenced in future to identify any possible mutations. Since RCMV ALL-03 is double stranded DNA virus, it was expected that the rate of mutation occurrence is extremely lower than RNA viruses which having higher rate of mutation incidence. This statement was supported by findings of this study where siRNAs treated RCMV ALL-03 having 100% sequence homology with stock of virus (progeny virus without siRNAs treated) proving the lower rate of mutation existence. The detailed information of aligned sequences for each siRNA treated regions compared with stock viruses were portrayed in Fig. 9.

## Discussion

While no approved vaccine or effective therapeutic treatment for CMV, great efforts on research are being made to treat the virus infection by different strategies [37–39]. The effort of making suitable vaccine for CMV started on early 2000 as reported by current National Academy of Medicine United States on the publication of a vaccine priority document [40]. Fascinatingly, CMV was given highest priority on the list and many strategies were implied to develop a suitable one by many biotechnology companies [41, 42]. Unfortunately, effective vaccines are still lacking and haven’t approved any by FDA. During the search of effective treatment for CMV, idea of controlling the disease by means of inhibiting virus replication and gene expression by combinations of two and more siRNAs had been explored in this study. Combination therapies have been tested before targeting other viral infections such as influenza, coronavirus, respiratory syncytial virus, hepatitis B&C, vaccinia virus [23, 25, 43–46]. It was hypothesized that targeting two different genes simultaneously could silence the expression of crucial viral genes thus; act as an antiviral therapy against CMV. There are many reasons to pick siRNA as a choice of potential treatment against CMV. First, they are easy to design and cheap to be produced [47]. Next, they are highly specific which can target almost any viral or cellular gene, and their stability can be increased by lyophilization [48]. An additional advantage was they can be delivered in combinations with many siRNAs or with other drugs targeting distinct regions in the same or different genes [46]. Undoubtedly, the mode of treatment action can be expanded by limiting the emergence of treatment resistance candidates [25]. Therefore, all these factors fueling up siRNA research and their successive clinical trials had increased remarkably in recent years [49–51].

Most of the treatments works fine on early stages found to be ineffective over certain period of time. This is due to the capability of viruses to develop resistance in order to escape the attack from antiviral therapies [52]. To resolve this, combination of siRNAs targeting similar or different gene region became popular approach of gene therapy. In detail, they could cleave multiple sites of mRNA which are troublesome for the target to get repaired. Silencing different crucial gene regions at a same time was expected to prevent the emergence of resistant viruses by lowering the possibilities of multiple mutation occurrences [12]. The effects of siRNAs could be enhanced by producing combinations pattern which can open a new window on gene therapeutics field. Therefore, the primary aim of this study is to investigate the suitability of combination of siRNAs acting as anti-CMV therapy in a proof of concept study. Although siRNA possess many advantages, unfortunately, the issue of siRNA’s off target effects still remain to be addressed and to deal with. Cytotoxicity assay was performed to rule out any combinations that having detrimental effects on the REF cell viability. This is because introducing foreign particles could elevate the level of cytotoxicity of cells which may end up in cell death. Unhealthy or dead cells are not able to sustain the virus production thus, can affects the results of siRNA effectivity [30]. Therefore, before the commencement of combinations siRNA effectivity assay, the cytotoxicity levels of the combinations were determined. The results displayed that these combinations siRNA except dpb + dpc + ie2b do not seem to induce a significant cytotoxicity level (*P* < 0.05) even at high concentration (300 pmol) in different time points. However, the cell viability for all the siRNAs was found to be constantly decreasing slightly over time. Two siRNA combinations (dpb + dpc, dpb + ie2b and dpc + ie2b) with each 150 pmol were found to be safe where cell viability was more than 70% at 72 h at high concentration (final 300 pmol). As expected, lower concentration starts from 25 pmol (each 12.5 pmol) to 3 pmol (each 1.5 pmol) was observed to have more than 90% of cell viability at 24, 48 and 72 h. However, quite high yet tolerable cytotoxicity level of three siRNA combination (dpb + dpc + ie2b) with each 100 pmol was encountered with cell viability of 50% at 300 pmol. Since 50% of cytotoxicity level is within the tolerable range, the three-siRNA combination (dpb + dpc + ie2b) was included in the subsequent siRNA experiments.

In the present study, combination of siRNAs targeting IE2 and DNA poly gene regions were proven to suppressing the viral gene expression and inhibiting the replication of virus. The four combinations of siRNA targeting same or different target regions of RCMV ALL-03 displayed effective inhibition of viral replication compared to scrambled siRNA, negative control non-targeting siRNA sample. Compared to GCV, a positive control of treatment group, combinations of duplex siRNAs: dpb + dpc, dpb + ie2b and dpc + ie2b showed better viral inhibition on day 10 and 18. However, combination of triple siRNAs: dpb + dpc + ie2b displayed lesser effective on controlling the viral replication compared to other group of treatments. The obtained results are in agreement with the relative quantification of gene expression by qPCR and also the rate of CPE formation.

Droplet digital PCR was employed to quantify the virus particles present in the treatment groups. Although it has the same role as real-time PCR, but this novel technology is a splendid adaptation of the current PCR being more convenient to be used as it is highly precise and sensitive, hence making results more reliable and easier to reproduce [53, 54]. More importantly, standard curve is not needed, and the results are interpreted directly by quantifying the droplets having the viral DNA particles. The quantification results revealed that least number of virus particles was observed for dpb + dpc treatment group followed by dpb + dpc, and dpb + dpc + ie2b, however, higher number was observed for dpc + ie2 treatment group. About 60% of viral DNA copies reduction was observed for dpb + dpc and dpb + ie2b while 42% for dpb + dpc + ie2b treatment group. Sadly, only 13% of viral DNA copies were reduced for dpc + ie2b presenting the least effective treatment group. From the detailed analysis, combination of siRNAs having dpb siRNA was found to perform better on virus inhibition rate compared to dpc and ie2b. The obtained results were proving the capability of dpb siRNA on inhibiting viral replication, which established as best siRNA candidate compared to others on individual assessment section previously. Except dpc + ie2b, other combination of siRNAs portrayed better inhibition on RCMV ALL-03 DNA compared to commercial drug GCV, which served as a positive control for treatment group. Thus, this justifies the need of alternate gene therapy treatment to control the CMV disease more effectively.

Naturally, hosts have many mechanisms to alert their immune system and activating the pathways to block the production of new progeny virus from spreading to other uninfected cells upon infection. To serve the purpose, apoptosis, is an innate cellular defense mechanism responsible to promote programmed cell death, consequently preventing the persistent infection. Apoptosis analysis revealed that combination siRNAs treated group shows a good number of early apoptosis compared to late apoptosis and necrosis cells of untreated control group. Out of four combinations of siRNAs, dpb + dpc have significant number of viable and early apoptotic cells compared to late apoptotic cells. Targeting DNA polymerase, dpb + dpc combination has successfully inhibiting the virus replication, thus blocking its capacity to suppress the apoptosis pathway. On the other hand, the other three combination siRNAs groups have similar pattern of results where most of the cells are accumulated on early apoptotic cells. No cells were identified on necrotic phase. As expected, majority of cells were observed under late apoptotic phase for negative control siRNA and untreated groups. Therefore, the combinations treatment proving the effectivity to suppresses the growth of RCMV ALL-03 which indirectly suppresses the apoptosis blocking pathway [55, 56]. Probably, combination siRNAs have good synergetic effect on maintaining the health of host cells.

Few observations were noticed where combination of siRNAs containing ie2b showed reduce level of IE2 gene expression compared to DNA poly. On the other hand, siRNAs containing dpb and dpc have better mRNA reduction level of DNA poly gene expression compared to IE2. For instance, dpb + dpc have high knockdown efficiency on DNA poly gene (79%) compared to IE2 (33%) since both candidates are targeting the same gene region. Our combination of siRNAs treatment are in agreement with the findings of individual siRNA where siRNAs targeting DNA poly gene region showed good reduction of mRNA level especially dpb and dpc siRNAs. Next, combination of triple siRNAs: dpb + dpc + ie2b showed much lower mRNA level (46–48%) reduction for both IE2 and DNA poly regions. This clearly showed that duplex siRNA combinations portrayed better inhibition rate of gene expression compared to triple siRNAs combination. The concept of more siRNAs targeting different regions could effectively inhibit virus replication and gene expression seems to be unworkable in our experiments targeting RCMV ALL-03.

Replication of virus in cell culture can be identified from distinctive morphologic changes of REF cell line designated as cytopathic effect also known as CPE [30]. Cytopathic action is commonly to giving us a clue whether the infectivity of virus is related to synthesis of infectious or noninfectious of virus particles. Thus, this can be a preliminary indicator for the RCMV ALL-03 identification purpose. The CPE analysis was carried out on day 14 pi. This is because approximately, it takes 8 to 10 days for CPE to starts and 90% CPE can be observed by day 14, as an agreement with CMV cell culture observation Loh et al. [30]. Lesser CPE rate was observed for dpb + dpc siRNAs treated group. The cells were started to enlarge, called as ballooning, which are very much better than the CPE conditions of GCV, negative control and untreated groups. Next, dpb + ie2b and dpc + ie2b combinations were displayed similar pattern of CPE with GCV control group, where plaques can be seen clearly due to the de-attachment of the REF cells caused by RCMV ALL-03 growth progression. Nevertheless, dpb + dpc + ie2b combinations showed to be less prominent to inhibit viral CPE rate where majority of the cells were infected, and the plaques are expanding bigger. Surprisingly, the CPE rate was about similar with untreated control group.

Achieving an effective antiviral therapy is much depended on low probability of drug resistance mutant development by an organism [57]. To identify the effects of custom designed combination of siRNAs effects on RCMV ALL-3 genome, the targeted region was sequenced and compared with stock virus. Remarkably, at 5 round of multiple siRNAs treatment, no any significant mutation was observed compared to original stock. This experiment does convey an important message regarding the importance of siRNA design approach. Specific siRNAs targeting highly conserved regions are capable to prevent or reduce the occurrence of mutant which can turn off the inhibitory effect of introduced siRNAs. Besides that, RCMV ALL-03 replication have been reduced by combination siRNAs treatment which indirectly limiting the likelihood of mutation occurrence by inhibiting the growth of virus population carrying the mutated gene for infection. In addition, CMV is double stranded DNA virus where the rate of mutation occurrence is low compared to other RNA viruses. Therefore, future studies on mutation identification need to be carried out at higher multiple round of siRNAs treatment.

In terms of performance, combination siRNAs containing IE2b especially dpb + ie2b and dpc + ie2b combinations displayed less efficient than dpb + dpc on inhibiting RCMV ALL-03 replication and gene expression. IE is the first ever gene to be transcribed after CMV infection. The IE products generated by differential splicing subsequently, will transactivate early genes. Initially, it was expected that silencing IE gene region particularly IE2 could stop the activation of other genes. Unfortunately, different scenario was observed where minimal expression of IE may adequate to turn on other genes, hence, progressing towards viral replication. This statement was supported by qPCR where high percentage of IE2 knockdown siRNAs yet expressing DNA polymerase and producing viral progeny.

Overall, according to the results obtained, combination of siRNAs found to possess antiviral activity against RCMV ALL-03, however, less impressive compared to highly efficient individual siRNAs when applied at same total concentration at 300 pmol. Unfortunately, the synergistic effects of combination siRNAs could not compete with the effectiveness of individual siRNAs from all the gathered results of virus titration, virus particles quantification, mRNA level investigation, CPE rate analysis and status of cell viabilities. The exact answer for the obtained results are unclear however, there are few reasons to explain the situation. First, there are higher chances for the siRNAs to compete each other for the association with RISC protein, which might have negative effects on each other’s synergistic activity [58]. Second, the proportion of each siRNA to get delivered into the cells is yet unclear even though the siRNAs were mixed at equal concentration. May one can get transported into the cells more often compared to other which could lead for the failure of synergistic effects. Next, the properties of siRNAs are varying among one another due to the different sequence composition, GC content, stability and affinity to bind the RISC protein [59]. More successful binding can direct the RISC to target so that more frequent cleavage to occur at particular target. On the other hand, least successful siRNAs binding to RISC would have lesser cleavage frequency in turn having lesser effectiveness. Thus, the individual characteristics of siRNA could be superior to another which the stronger siRNA have more silencing effects compared to weaker one [60].

Some studies have tried combination siRNAs for the purpose of silencing viral gene target and cellular host factor which support the virus replication. For example, another study has been conducted and proved that HCV replication was inhibited pronouncedly by introducing siRNAs which are targeting RNA dependent RNA polymerase viral gene as well La autoantigen of cellular factor [23]. Therefore, similar perspective of experiment can be conducted for RCMV ALL-03 in future to maximize the efficiency of combination siRNAs on inhibiting the virus replication and gene expression. However, one needs to be more careful on choosing the cellular factor where the effects of silencing don’t bring any detrimental harm for the host [61]. Taken all these together, combinations siRNAs application found to be beneficial in inhibiting the virus replication and more research need to be conducted to enhance the efficiency. Despite human viruses, this system can be utilized for animal viruses which having an unbearable impact on poultry industries due to virus infections [60, 62]. Apart from that, this combinatorial siRNAs approach could replace as an alternate treatment especially for plant-based vaccines which encountering difficulties during production [63].

## Conclusion

This is the first study initiated to investigate the effectiveness of siRNA combinations to inhibit Malaysian isolate CMV. The combinations were formulated based on the individual performance on inhibiting RCMV ALL-03. The results markedly inhibited by viral genomic DNA, gene expression and CMV replication in vitro, however, the findings are varying according to the combinations. The siRNA combinations containing dpb siRNA showed better rate of viral inhibition, as similar with the individual characteristics. Overall, combinations having two siRNAs showed promising results that the triple siRNA combination. Interestingly, no any mutation occurrence was encountered among the treated groups thus highlighting its feasibility to be used as treatment option in future.

## Supplementary information

**Additional file 1: Figure S1.** Standard curve and amplification curve of the siRNAs and GAPDH were determined from tenfold serial dilution of RNA isolated from treated cells. **Figure S2.** The cytotoxicity of combinations siRNA targeting RCMV ALL-03 and negative control siRNA in REF cells. Cells were transfected with lipofectamine 3000 and siRNA complex, which were prepared in final concentration of 300 pmol of siRNA and incubated for 6 h and an additional of 24/48/72 h at 37℃. The cellular viability was measured by MTT assay. Each treatment was performed in triplicate and repeated in two independent experiments. Results represent Mean ± Standard deviation. **Figure S3.** Gating references used for unstained, PI and FITC apoptosis analysis using flow cytometry. **Figure S4.** 1D ddPCR analysis plot shows detection of RCMV ALL-03 DNA in siRNA treated and non-treated groups. Each sample was partitioned into an average of 10,000 droplets per well and replicated in two wells. The droplet counts for positive (blue) and negative (gray) from all replicated wells were combined to yield a “merged” well. A fluorescence amplitude value > 1176 (pink line) was considered to be a positive droplet.

## Data Availability

All data generated or analyzed during this study are included in this manuscript.

## Abbreviations

siRNA: Small interfering RNA
CMV: Cytomegalovirus
RCMV: Rat Cytomegalovirus
IE2: Immediate early 2
pmol: Picomole
CPE: Cytopathic effect
ddPCR: Droplet digital PCR
