# Supplementary material for: Multiple gene targeting siRNAs for down regulation of Immediate Early-2 (Ie2) and DNA polymerase genes mediated inhibition of novel rat Cytomegalovirus (strain All-03)
Source: Virol J. 2020 Oct 27;17:164. doi: 10.1186/s12985-020-01436-5 (PMC7590257; doi:10.1186/s12985-020-01436-5)
Supplement: Supplementary file 1 — Additional file 1: Figure S1. Standard curve and amplification curve of the siRNAs and GAPDH were determined from tenfold serial dilution of RNA isolated from treated cells. Figure S2. The cytotoxicity of combinations siRNA targeting RCMV ALL-03 and negative control siRNA in REF cells. Cells were transfected with lipofectamine 3000 and siRNA complex, which were prepared in final concentration of 300 pmol of siRNA and incubated for 6 h and an additional of 24/48/72 h at 37℃. The cellular viability was measured by MTT assay. Each treatment was performed in triplicate and repeated in two independent experiments. Results represent Mean ± Standard deviation. Figure S3. Gating references used for unstained, PI and FITC apoptosis analysis using flow cytometry. Figure S4. 1D ddPCR analysis plot shows detection of RCMV ALL-03 DNA in siRNA treated and non-treated groups. Each sample was partitioned into an average of 10,000 droplets per well and replicated in two wells. The droplet counts for positive (blue) and negative (gray) from all replicated wells were combined to yield a “merged” well. A fluorescence amplitude value > 1176 (pink line) was considered to be a positive droplet. [file 12985_2020_1436_MOESM1_ESM.docx]

**Figure S1: Standard Curve generation for qRT-PCR**

Taqman qRT-PCR Standard Curve Generation of Housekeeping Gene GAPDH


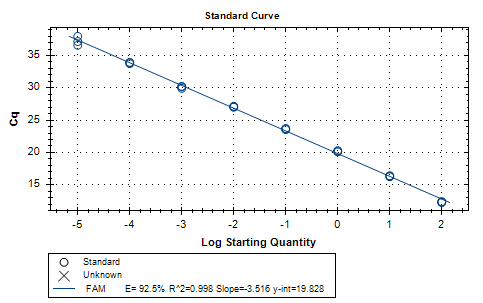


Taqman qRT-PCR Amplification Curve Generation of Housekeeping Gene GAPDH


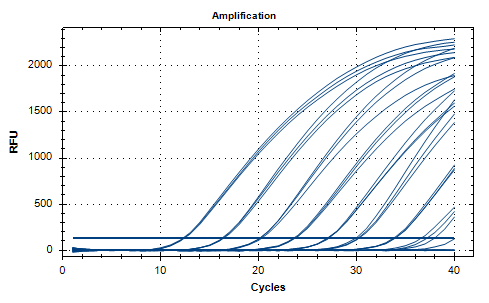


Taqman qRT-PCR Standard Curve Generation of Ie2b siRNA


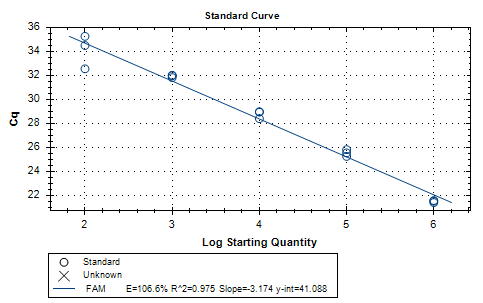


Taqman qRT-PCR Amplification Curve Generation of Ie2b siRNA


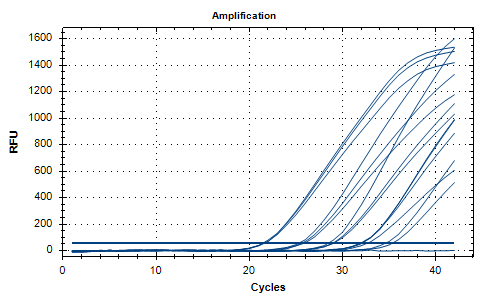


Taqman qRT-PCR Standard Curve Generation of dpb siRNA


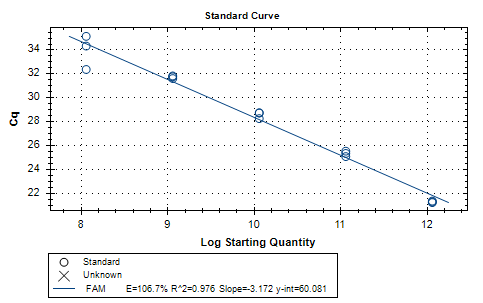


Taqman qRT-PCR Amplification Curve Generation of dpb siRNA


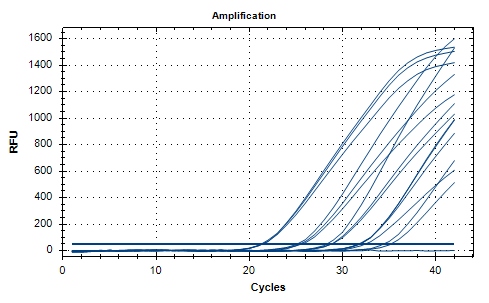


Taqman qRT-PCR Standard Curve Generation of dpc siRNA


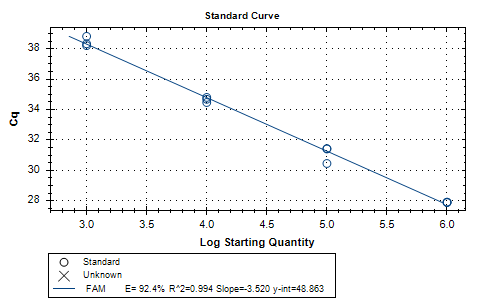


Taqman qRT-PCR Amplification Curve Generation of dpc siRNA


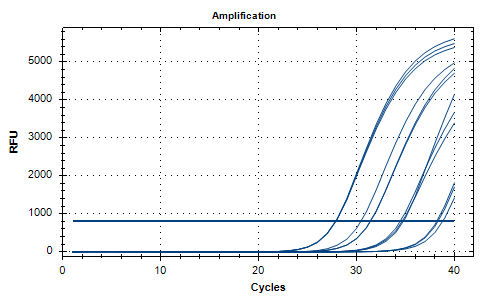


Figure S1: Standard curve and amplification curve of the siRNAs and GAPDH were determined from 10 fold serial dilution of RNA isolated from treated cells.

**Figure S2: Cytotoxicity of combination siRNAs**

**
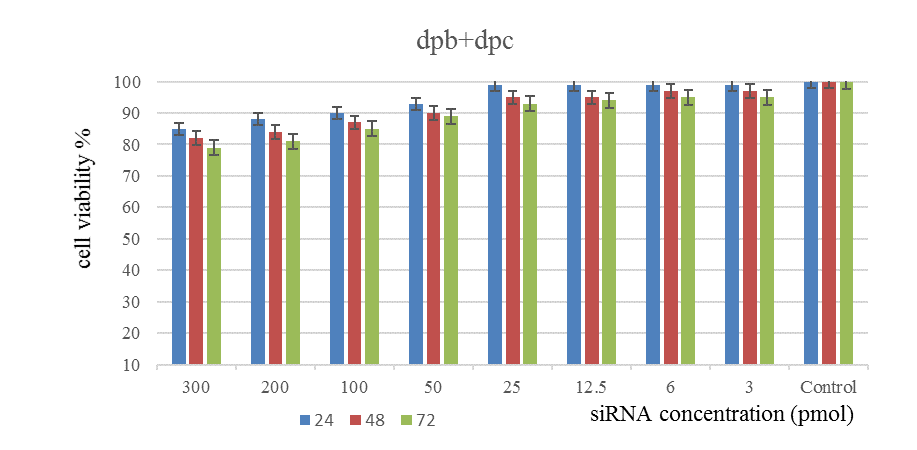
**

**
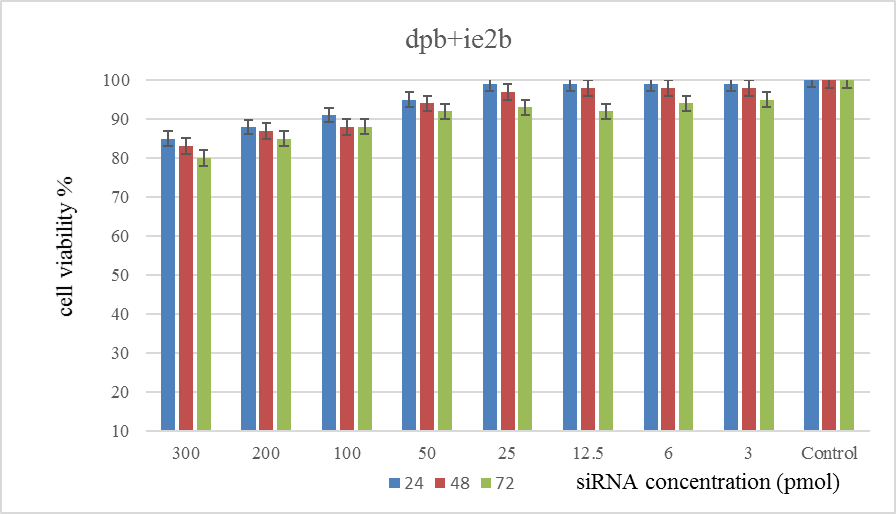
**

**
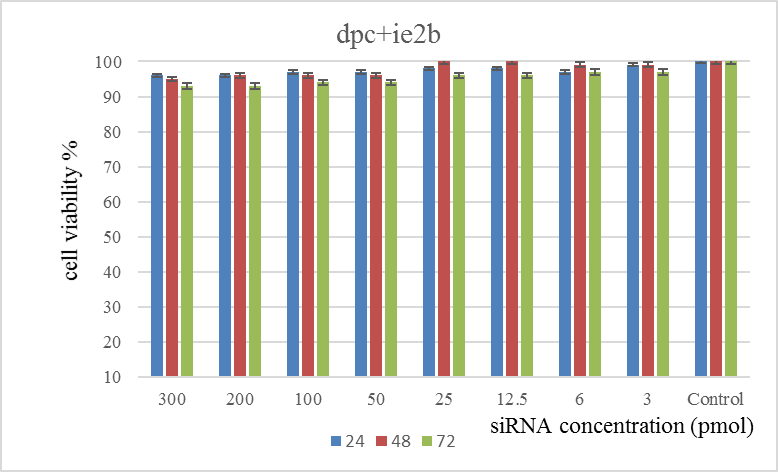
**

**
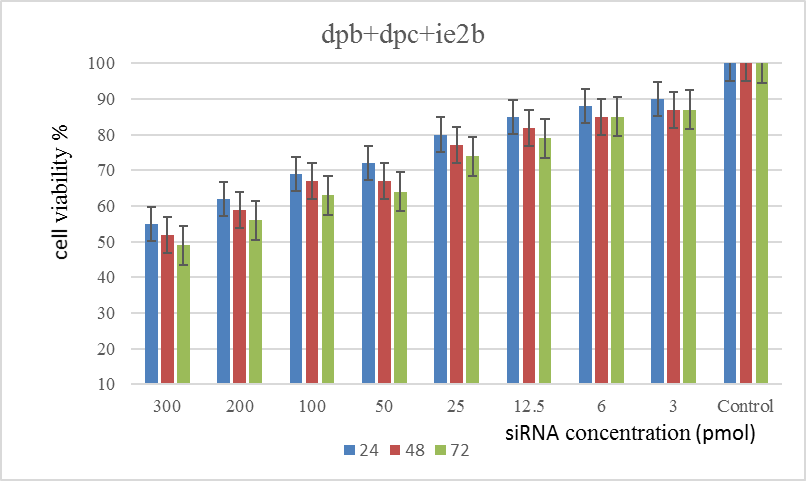
**

Figure S2: The cytotoxicity of combinations siRNA targeting RCMV ALL-03 and negative control siRNA in REF cells. Cells were transfected with lipofectamine 3000 and siRNA complex, which were prepared in final concentration of 300 pmol of siRNA and incubated for 6 hours and an additional of 24/48/72 hours at 37℃. The cellular viability was measured by MTT assay. Each treatment was performed in triplicate and repeated in two independent experiments. Results represent Mean ± Standard deviation.

**Figure S3: Gating reference for UNSTAINED for apoptosis analysis**


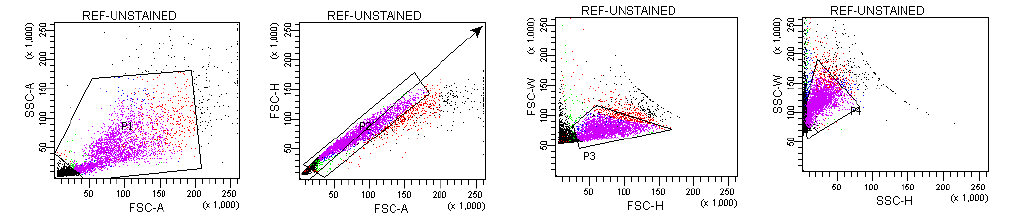


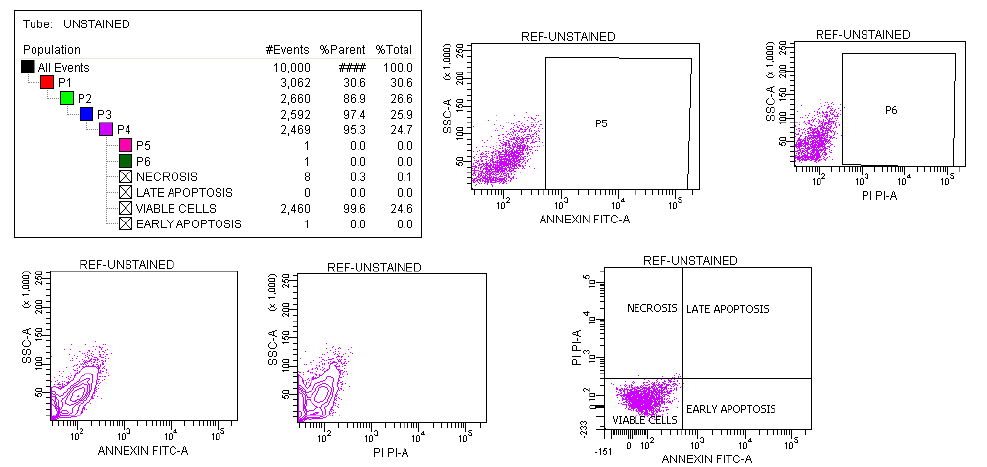


Gating reference for PI only for apoptosis analysis


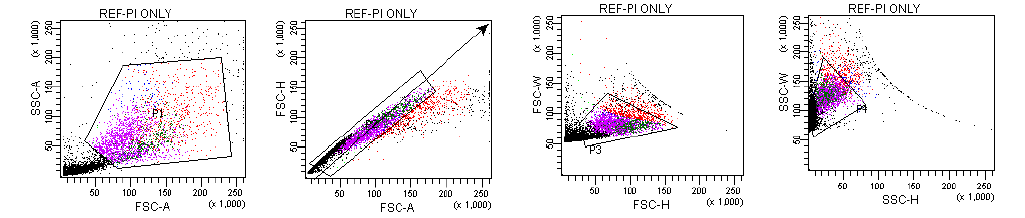


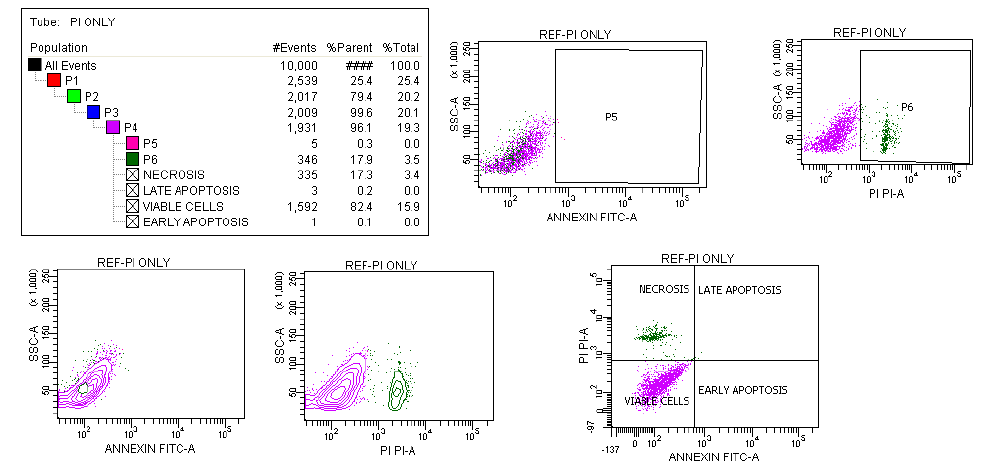


Gating reference for FITC only for apoptosis analysis


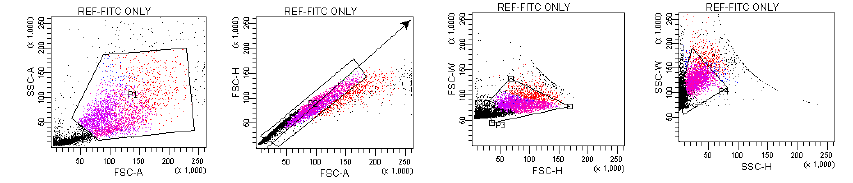


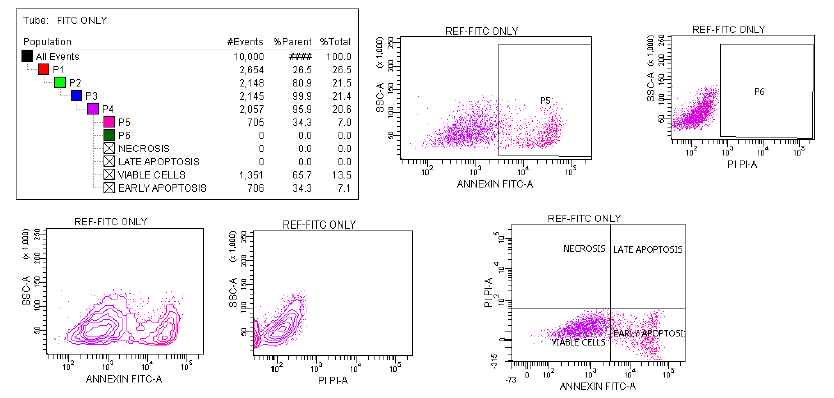


Figure S3: Gating references used for unstained, PI and FITC apoptosis analysis using flow cytometry.

**Figure S4: 1D ddPCR analysis plot**


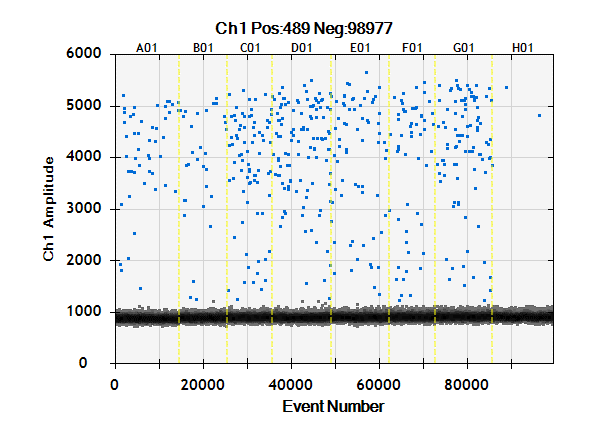


Figure S4: 1D ddPCR analysis plot shows detection of RCMV ALL-03 DNA in siRNA treated and non-treated groups. Each sample was partitioned into an average of 10,000 droplets per well and replicated in two wells. The droplet counts for positive (blue) and negative (gray) from all replicated wells were combined to yield a “merged” well. A fluorescence amplitude value >1176 (pink line) was considered to be a positive droplet.
